# Editorial: Achieving Efficient Diabetes Care Through Understanding the Risk Factors, Markers, and Patient Experiences

**DOI:** 10.3389/fendo.2022.854167

**Published:** 2022-03-30

**Authors:** Boon-How Chew, Rimke C. Vos, Indah Suci Widyahening, Kamlesh Khunti

**Affiliations:** ^1^ Department of Family Medicine, Faculty of Medicine and Health Sciences, Universiti Putra Malaysia, Serdang, Malaysia; ^2^ Clinical Research Unit, Hospital Pengajar Universiti Putra Malaysia (HPUPM Teaching Hospital), Persiaran MARDI - UPM, Serdang, Malaysia; ^3^ Department Public Health and Primary Care/Leiden University Medical Center (LUMC)-Campus The Hague, Leiden University Medical Center, Hague, Netherlands; ^4^ Department of Community Medicine, Faculty of Medicine Universitas Indonesia, Jakarta, Indonesia; ^5^ National Institute for Health Research Applied Research Collaboration-East Midlands, Leicester Diabetes Centre, Leicester, United Kingdom; ^6^ Diabetes Research Centre, Leicester General Hospital, University of Leicester, Leicester, United Kingdom

**Keywords:** diabetes care, efficient, risk factors, biomarkers, patient’s experience

Until recently, the concept of a world without diabetes mellitus (DM) was not possible. This is now foreseeable with therapeutic advancements in technology in the form of our ability to create an artificial pancreas, stem cell transplant, personalized medicine of genomic-guided treatments, metabolic surgery, lifestyle interventions (1), and nutraceutical medicine (2). The greater challenges in delivering these therapies require adaptable healthcare systems, a trained healthcare workforce, and accessible and affordable treatments for those in greatest need of it. Bridging the gap between now and a better future for diabetes management requires a collective global effort that crosses international borders and boundaries, and the need for such a development is continuously pressing and supported by all organisations and governments. This begins by achieving an understanding of the increasing burden (3) and challenges of DM and its related complications when it comes to the healthcare systems, healthcare workers, and the person-level risk factors of DM. But this is also true for the markers of diseases related to DM, such as the life experiences of the people living with DM in coping and coming to terms with the disease in the form of a therapeutic relationship with professional careers and healthy living (4). The latter also requires local studies for more direct evidence and contexts (Al-Rifai et al., Sunny et al., Bukhsh et al. and de Jong et al.). In this special Frontiers in Endocrinology Research Topic, a number of articles examine this and the biological evidence of the maternal fasting plasma glucose profile in early pregnancy and its effect on foetal growth and birth outcomes (Guo et al.), preterm birth and birth weight, and the risk of type 1 diabetes (T1D) (Huang et al.), diabetes and sarcopenic obesity (Wang et al.), sodium-glucose cotransporter-2 inhibitors on liver enzyme (Euh et al.), and abnormalities in non-coding RNA as biomarkers (Chi et al.). This collective evidence takes us a step closer to the realisation of effective diabetes care for the prevention and normalisation of DM.

Due to gestational diabetes mellitus (GDM), a new generation of human beings is set to be at higher risk of DM (Guo et al., Huang et al.). Women with GDM have almost a 10 times higher risk for type 2 diabetes (T2D) compared to healthy women (5). Therefore, studying and reporting the prevalence and risk factors for GDM in the Middle East and North Africa (MENA) region is timely. Al-Rifai et al. have conducted a systematic review of 102 articles including 279,202 pregnant women from 16 countries in the MENA region: Algeria (1 included article), Bahrain (2), Egypt (4), Iraq (3), Iran (37), Jordan (4), Lebanon (2), Libya (1), Morocco (1), Oman (5), Qatar (6), Saudi Arabia (22), Sudan (3), Tunisia (1), United Arab Emirates (UAE) (9), and Yemen (1). This study reports a relatively high prevalence of GDM, ranging from 4.7% (95% CI, 3.0-6.7%; six studies) in Jordan to 20.7% (95% CI, 15.2-26.7%; 19 studies) in Qatar, giving an overall pooled weighted GDM prevalence in the MENA region of 13.0% (95% CI, 11.5-14.6%) in the period of 2000–2019. Pregnant women aged ≥30 years (adjusted OR 2.5, 95% CI, 1.5-4.2) and obese pregnant women (aOR 2.9, 95% CI, 1.5-5.7) were significant predictors of GDM. The wide variation in the prevalence rates in a number of published systematic reviews is due to the use of different GDM diagnostic criteria in the included studies. In another linked article, Guo et al. report data on 35,981 women (70% primigravidae); they show that a per-unit increase in the first trimester (9–14 weeks) fasting plasma glucose (FPG) levels was negatively associated with foetal growth parameters in mid pregnancy (18-24 weeks) but positively correlated with those in late pregnancy (28–34 weeks) and with birth characteristics (large-for-gestational age). The effect of first-trimester FPG levels on foetal weight (and preterm birth) was no longer of statistical significance after additional adjustment for pre-pregnancy BMI. However, this effect was present in mothers who were older (35 years), had a family history of diabetes, and had multiparity. Importantly, the study showed in the sensitivity analyses that the negative relationship between maternal FPG and foetal growth in mid pregnancy and the positive relationship in late pregnancy were essentially similar in pregnant women without GDM, and in pregnancy without common medical problems gestational hypertension, preeclampsia, placenta previa, placental abruption and cholestasis of pregnancy. Another study from China by Huang et al. suggests that T1D could be more related to preterm birth (<37 weeks) and less to birth weight, except for Chinese girls with a high birth weight (OR 3.2, 95% CI 1.3-7.5).

Efficient diabetes screening for women with a history of GDM can be hindered by inappropriate self-perceived future risk of T2D as well as many other personal, socioeconomic, and healthcare system-level barriers. These factors reported by Sunny et al. from Singapore (from both women who did and did not take up screening) are not unexpected to many, but it is remarkable how unconcerned many potential social supporters of these women and inaccessible healthcare services can be when it comes to postpartum diabetes screening for these high-risk women. There are very similar personal and psychosocial barriers to self-care among adults with T2D in Lahore, Pakistan (Bukhsh et al.). From the perspectives of human health behaviours, this shows the complexity and interplay of the (intrinsic) psychological (value system, emotion, and cognition) and the (extrinsic) sociological and environmental factors that we see in people living with DM. The intrinsic factors (knowledge, skills, and motivation) are believed to be the key strengths most worthwhile for the implementation of interventions to lower the risk of DM (6). This intrinsic and personal quality is possibly enhanced when extrinsic factors are facilitated, such as having an engaged family and members of your social circle, a healthy diet and good food quality, conducive places for activity, and the experience of readily available drugs and affordable healthcare. In terms of diabetes care specific to cardiovascular risk assessment and screening for diabetes-related complications, de Jong et al. show in their systematic review that men may be less likely to receive retinopathy screenings and women less likely to receive foot exams. This gender disparity in terms of the uptake of routine screening services is likely to be more obvious in lower economic countries, and this poses another challenge to efficient diabetes care that will require creative solutions based on a good understanding of the local context of the people, informed healthcare workers, and genuine involvement from policymakers and pertinent stakeholders.

Finally, Wang et al., Euh et al., and Chi et al. provide further interesting evidence on the clinical sciences of sarcopenic obesity, the effect of sodium-glucose cotransporter-2 inhibitors on weight and the liver, and the potential roles of non-coding RNAs (ncRNAs) in precision medicine of people with DM, respectively. Sarcopenia and obesity in diabetes and ncRNAs are emerging topics that may be worth further attention from researchers that study DM in the quest for a more efficient and effective diabetes care strategy. By bringing all these studies together in this special Research Topic, we hope the linked findings will help to develop and implement healthcare and policy strategies to reduce disparities that occur due to the wider determinants of health.

## Author Contributions

BC drafted the editorial. KK commented and contributed critically, RV and IW commented and further improved the editorial. All agreed to the version to be submitted.

## Funding

KK is supported by the National Institute for Health Research (NIHR) Applied Research Collaboration East Midlands (ARC EM) and the NIHR Leicester Biomedical Research Centre (BRC).

## Conflict of Interest

KK has acted as a consultant, speaker or received grants for investigator-initiated studies for Astra Zeneca, Novartis, Novo Nordisk, Sanofi-Aventis, Lilly and Merck Sharp & Dohme, Boehringer Ingelheim, Bayer, Berlin-Chemie AG/Menarini Group, Janssen, and Napp.

The remaining authors declare that the research was conducted in the absence of any commercial or financial relationships that could be construed as a potential conflict of interest.

## Publisher’s Note

All claims expressed in this article are solely those of the authors and do not necessarily represent those of their affiliated organizations, or those of the publisher, the editors and the reviewers. Any product that may be evaluated in this article, or claim that may be made by its manufacturer, is not guaranteed or endorsed by the publisher.
